# Functional outcome in older adults with joint pain and comorbidity: design of a prospective cohort study

**DOI:** 10.1186/1471-2474-12-241

**Published:** 2011-10-24

**Authors:** Lotte AH Hermsen, Stephanie S Leone, Daniëlle AWM van der Windt, Martin Smalbrugge, Joost Dekker, Henriëtte E van der Horst

**Affiliations:** 1 Department of General Practice and the EMGO Institute for Health and Care Research, VU University Medical Center, Amsterdam, The Netherlands; 2 Arthritis Research UK, Primary Care and Health Sciences, Keele University, Keele, Staffordshire, UK; 3 Department of Nursing Home Medicine and the EMGO Institute for Health and Care Research, VU University Medical Center, Amsterdam, The Netherlands; 4 Department of Rehabilitation Medicine and the EMGO Institute for Health and Care Research, VU University Medical Center, Amsterdam, The Netherlands

## Abstract

**Background:**

Joint pain is a highly prevalent condition in the older population. Only a minority of the older adults consult the general practitioner for joint pain, and during consultation joint pain is often poorly recognized and treated, especially when other co-existing chronic conditions are involved. Therefore, older adults with joint pain and comorbidity may have a higher risk of poor functional outcome and decreased quality of life (QoL), and possibly need more attention in primary care. The main purpose of the study is to explore functioning in older adults with joint pain and comorbidity, in terms of mobility, functional independence and participation and to identify possible predictors of poor functional outcome. The study will also identify predictors of decreased QoL. The results will be used to develop prediction models for the early identification of subgroups at high risk of poor functional outcome and decreased QoL. This may contribute to better targeting of treatment and to more effective health care in this population.

**Methods/Design:**

The study has been designed as a prospective cohort study, with measurements at baseline and after 6, 12 and 18 months. For the recruitment of 450 patients, 25 general practices will be approached. Patients are eligible for participation if they are 65 years or older, have at least two chronic conditions and report joint pain on most days. Data will be collected using various methods (i.e. questionnaires, physical tests, patient interviews and focus groups). We will measure different aspects of functioning (e.g. mobility, functional independence and participation) and QoL. Other measurements concern possible predictors of functioning and QoL (e.g. pain, co-existing chronic conditions, markers for frailty, physical performance, psychological factors, environmental factors and individual factors). Furthermore, health care utilization, health care needs and the meaning and impact of joint pain will be investigated from an older person's perspective.

**Discussion:**

In this paper, we describe the protocol of a prospective cohort study in Dutch older adults with joint pain and comorbidity and discuss the potential strengths and limitations of the study.

## Background

Almost half of the community dwelling older adults report daily pain [1], which mostly concerns pain in muscles and joints [2]. Joint pain often affects functioning, in terms of mobility, functional independence, participation in social activities, as well as quality of life (QoL) [3-6], and is among the ten leading causes of disability-adjusted life years in high income countries [7]. In daily clinical routine, the general practitioner (GP) is the first point of contact for older adults with joint pain and provides both assessment and treatment [8]. However, evidence suggests that only 15-30% of the older population with joint pain consult their GP [9-14], despite several available treatment options. Furthermore, research shows that joint pain is often poorly recognized and treated in primary care when older people do contact their GP [2,12,15-19].

Poor recognition and treatment of joint pain is especially seen in older patients who also suffer from other chronic conditions, such as diabetes, cardiovascular disorders or respiratory diseases [16,20,21] and suggests that the presence of comorbid chronic conditions complicates appropriate recognition, assessment and management of joint pain. As the prevalence of co-existing chronic conditions with joint pain is reported to be between 65-85% in the older population [19,22], this could represent an important problem in primary care. The observed suboptimal care for patients with joint pain and the relation of both joint pain and other chronic conditions with disability and impairment [18,19], indicate that older adults with joint pain and comorbidity have a higher risk of poor functional outcome and decreased QoL and may benefit from more effective management in primary care.

To optimize health care for this population, it could be relevant for health care providers to identify older adults at risk of poor functional outcome and decreased QoL. Early recognition of those at risk may facilitate better targeting of treatment, resulting in more effective and efficient health care for older adults with joint pain and comorbidity. To enable early recognition of poor functional outcome, it is important to obtain more insight in functioning and the course of functioning in the defined group. This provides the opportunity to make an appropriate distinction in subgroups based on functional prognosis and to understand the differences in functioning in older adults with joint pain and comorbidity. It also helps to identify possible risk factors that are associated with different patterns of functioning and therefore makes it possible to predict specific trajectories of functioning.

Previous longitudinal studies on prognostic factors for functional decline in joint pain and/or osteoarthritis (OA) found evidence for various predictors of poor functional outcome, like older age, high pain intensity, longer duration of symptoms, comorbidity, high BMI, anxiety, depression and poor self efficacy [23-26]. However, this evidence is limited because the majority of the studies focused on one particular type of joint pain, on different age groups or did not deal adequately with comorbidity. Furthermore, these studies were especially interested in physical functioning and lacked information about the role of joint pain on aspects of social functioning such as participation and functional independence, which are indicated as important outcomes for older people with joint pain [4,6,14,27]. This implicates the need for further research on different aspects of functioning in older adults with joint pain and comorbidity, that highlights both physical functioning and social functioning.

Apart from investigating functional outcomes, it is also important to obtain insight into health care utilization and health care needs in older people with joint pain and comorbidity [9,12,28] and to investigate the personal experiences and impact of joint pain in everyday life. Broad exploration will provide information about the various strategies older people use to manage their pain and barriers and opportunities in the care for older adults with joint pain and comorbidity, which will help to further optimize health care for this defined group.

### Objectives

The overall aim of the study is to explore functioning in older adults with joint pain and comorbidity, in terms of mobility, functional independence and participation and to identify possible predictors of poor functional outcome. The results will be used to develop prediction models for the identification of subgroups at high risk of poor functional outcome. Besides identifying predictors of functioning, we will also explore QoL and its possible predictors. Furthermore, the study examines health care use and health care needs in this population and explores the personal experiences and impact of joint pain in everyday life, from an older person's perspective.

## Methods/Design

### Study design

The study has been designed as an observational prospective cohort study, with measurements at baseline and after 6, 12 and 18 months. Various methods will be used to gather information, using questionnaires, physical tests, patient interviews and focus groups.

The Medical Ethics Committee of the VU University Medical Center, Amsterdam, has approved the study protocol.

### Study population

Patients will be recruited from approximately 25 general practices, located in the Northwest of the Netherlands. Patients are eligible for participation in the study if they are aged 65 or older, have two or more chronic conditions (listed in table 1) next to osteoarthritis or other musculoskeletal pain conditions, report joint pain on most days during the last month and give informed consent. Patients will be excluded from participation if they live in a nursing home, reside in a foreign country or outside the research area for prolonged periods of time, have a life threatening illness or short life expectancy (terminally ill), suffer from serious cognitive impairment/dementia, or have insufficient knowledge of the Dutch language.

**Table 1:** Chronic conditions for inclusion, based on a list of chronic conditions defined by the CBS

Chronic conditions	ICPC codes
Pulmonary disease/chronic respiratory disease	R91 R95, R96
Chronic ischemic heart disease, heart failure	K73, K74, K75, K76, K77, K78, K79, K82, K83, K84
Peripheral arterial heart disease (atherosclerosis)	K91, K92
Cerebrovascular disease (stroke, TIA)	K89 K90
Diabetes Mellitus	T90
Chronic thyroid disorder	T85, T86
Nervous system disorder (multiple sclerose, parkinson's disease, epilepsy)	N86, N87, N88
Vertigo/dizziness	N17
Colitis ulcerosa	D94
Urinary incontinence	U04
General disability, handicap	A28
Visual disturbances/loss	F28, F84, F93, F94
Hearing disturbances/loss	H28, H84, H86
Memory, concentration, orientation impairment	P20
Psychoses/schizophrenia	P71, P72, P73 en P98
Anxiety disorder	P74
Depression	P76
Malignant tumors	A79, B72, B73, B74, D74, D75, D76, D77, F74, H75, K72, N74, R84, R85, S77, T71, T73, U75, U76, U77, U79, X75, X76, X77, X81, Y77, Y78

### Selection procedure

**Figure 1:**
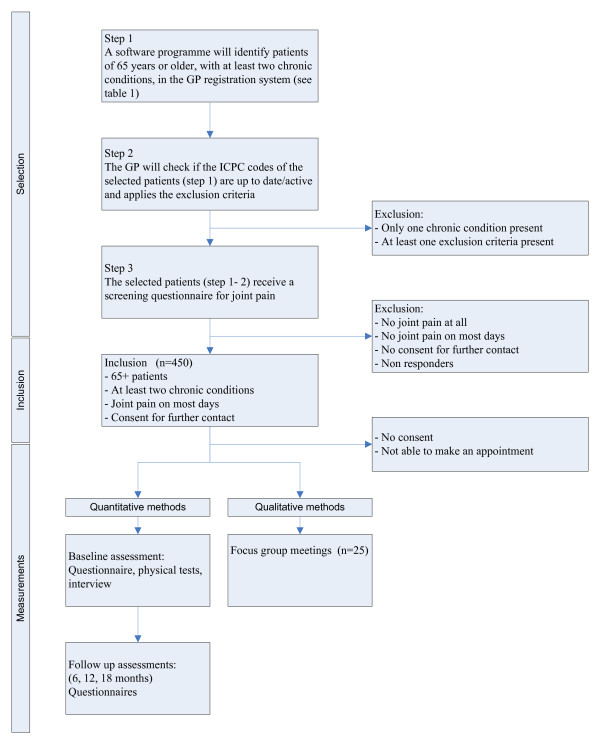
**Flow chart for selection, inclusion and data collection**.

The selection procedure will be conducted in three steps. The first step consists of the identification of patients of 65 years and older with at least two chronic conditions in the participating general practices. In the second step, the general practitioner will check if the chronic conditions of the selected patients are still up-to-date and will apply the exclusion criteria. In the third step, all of the patients selected in the previous steps, will be screened for the presence of joint pain, by using a questionnaire. The steps in the selection procedure are explained below, and summarized in Figure 1.

#### Step 1

General practices use a computerized registration system, in which symptoms or diagnoses are classified, according to the International Classification of Primary Care (ICPC codes) [29]. When complains are recurrent, chronic, or have lasting consequences for functioning, the ICPC codes are registered in the so-called problem list. We will use specially developed software to search the GP registration system for patients who meet the inclusion criteria of being aged 65 or older and having at least two chronic conditions (table 1), as coded in the problem list. The programme will automatically exclude patients with an ICPC code for dementia (P70), as cognitive impairment is an exclusion criterion for participation.

#### Step 2

**Figure 2:**
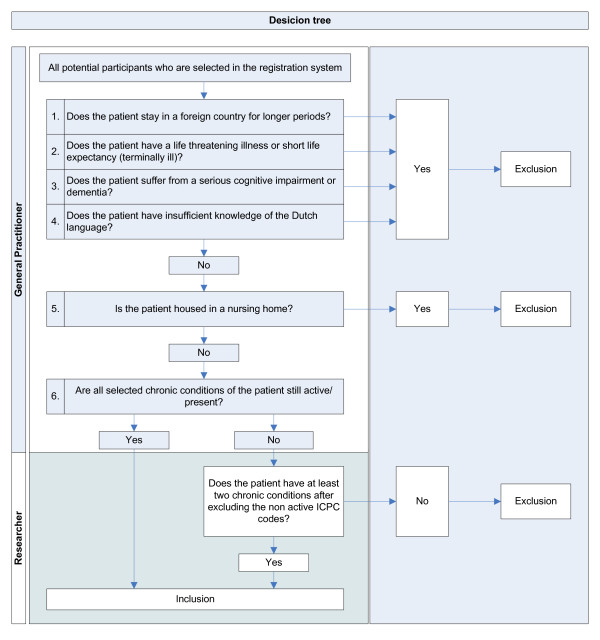
**Decision tree that general practitioners can use during step 2 of the inclusion procedure**.

ICPC codes of symptoms or conditions that have been resolved are retained in the registration system of the general practitioner. If we would select patients based on ICPC codes of resolved conditions, this may lead to an incorrect selection in step 1. Therefore, in the second step, we ask the general practitioner to check if the selected chronic conditions of the patients are still up-to-date and active. A chronic condition is considered 'active' if it has the attention of the general practitioner, which for example involves additional diagnostic testing or monitoring, recent treatment or medication prescription, or if the condition is known to have a progressive course [30]. Subsequently, we will ask the general practitioner to exclude patients who meet one or more of the above mentioned exclusion criteria. We developed a tool to facilitate the general practitioners during this check. This so-called "decision tree" is shown in Figure 2 and consists of six questions that helps the GP to screen all patients for non-active chronic conditions and the exclusion criteria.

#### Step 3

The third step is to send a study information leaflet, information letter and self-report screening questionnaire to all potential participants identified in the previous two steps. This screening questionnaire contains questions about the presence, frequency, duration and intensity of joint pain and also assesses the impact of joint pain on functioning. Additionally, patients are asked to consent to further contact. Reminders will be sent to non-responders after two weeks. Responders who report experiencing joint pain on most days in the last month and give permission to be re-contacted will be included in the study (Figure 1).

### Measurements

Measurements will be performed at baseline and after 6, 12 and 18 months. We will use various methods to collect data, i.e. questionnaires, physical tests, interviews and focus groups. To optimize the data collection, we have invited two older adults who represent the target population, to participate in our project team as patient experts. They will be asked to assess the information leaflet, letters and questionnaires for clarity and burden to patients and to give recommendations about relevant topics that we can incorporate in the focus group meetings.

### Procedure

The baseline assessment will consist of a baseline questionnaire, several physical tests and an interview. The physical tests and interview will be performed at the participant's home, during a home visit. The baseline questionnaire will be sent to the participant's home two weeks before the visit, so that the participant has enough time to complete the questionnaire. An interviewer will contact eligible respondents by telephone to provide information about the project, to explain the procedure for the baseline assessment in more detail and to schedule an appointment for the baseline assessment. During the visit, written informed consent will be obtained. Then, the interviewer will conduct the short set of physical tests (as described in table 2) and the interview by using the Camberwell Assessment of Needs for Elderly People (CANE) [31]. Finally, the interviewer will collect and check the baseline questionnaire, which will conclude the visit.

**Table 2:** Baseline and follow-up measurements

Outcome	Method	Data*source	At baseline	At 6 months	At 12 months	At 18 months
**Functional outcomes**						
Mobility	Short Form-36 Health Survey (SF-36): subscale on physical functioning [34,35]	Q	x	x	x	x
Participation	Keele Assessment of Participation (KAP). Person-perceived, performance based participation [37]	Q	x	x	x	x
Functional independence	Katz Index of independence in activities of daily living (KATZ) [36]	Q	x	x	x	x

**Quality of life**						
QoL	One item of the SF-36. Cantril's self anchoring ladder [38]	Q	x	x	x	x

**Possible predictors of functioning and QoL**						
Pain	Chronic Pain Grade (CPG), domains on pain intensity and disability [47] Items measuring frequency, duration and intensity of pain [48]	Q	x	x	x	x
Comorbidity	Presence of various illness (yes/no)	Q	x		x	
Physical performance	Short Physical Performance Battery Score: balance (tandem stance), timed 6 m walk, 5x chair stands [49]	P	x			
Frailty	Unintentional weight loss: Based on BMI [50]	P	x			
	Exhaustion: One item from the SF-36 *("how much of the time in the past 4 weeks did you have a lot of energy?*") [50]	Q	x			
	Weakness: Measuring grip strength [51]	P	x			
	Slowness: Measuring walking speed [51]	P	x			
	Low physical activity: Physical Activity Scale for the Elderly (PASE) [50,52,53]	Q	x			
Utility	EQ-5D+c. Measures health outcome: mobility self-care, usual activities, pain, anxiety/depression and cognition [54]	Q	x		x	
Functional status	Short Form-36 Health Survey: domains on physical functioning, social functioning and role limitations [35]	Q	x	x	x	x
Well being	Short Form-36 Health Survey: domains on mental health, vitality and pain [35]	Q	x	x	x	x
Overall health	Short Form-36 Health Survey: domains on general health perception and health change [35]	Q	x	x	x	x
Anxiety and depression	Hospital Anxiety and Depression Scale (HADS) [55]	Q	x	x	x	x
Personal control	Brief Illness Perception Questionnaire (B-IPQ) [56]	Q	x	x	x	x
Coping with pain	Two- item Coping Strategies Questionnaire (CSQ) [57,58]	Q	x	x	x	x
	Pain Coping Inventory (PCI): subscale resting (5 items) [59]	Q	x	x	x	x
Self-efficacy	Arthritis Self Efficacy Scale (ASES) [60]	Q	x	x	x	x
Mobility outside the home	Access to material goods and services (car, public transport, GP, chemist, internet) and living environment, residential.	Q	x			
Social isolation and perceived social support	Social Support Scale (SOS) [61]	Q	x			
Body Mass Index	Based on height and weight, measured by a standardized protocol	P	x			
Falls	Items on past falls					
Life style factors	Items on smoking behaviour and alcohol consumption	Q	x			
Sociodemographic characteristics	Age, gender, ethnicity, living arrangements, ZIP code, marital status, education, employment status	Q	x		x	

**Health care utilization and health care needs**						
General health care use	6-items(yes/no): hospital admissions, unplanned GP visits, homecare, temporary admission nursing or care home, day care and day treatment	Q	x		x	
Health care use for joint pain	Current use of pain medication, creams, gels or braces. Participation in exercise programmes.	Q	x			

* Q = Questionnaire, P = Physical assessment

During the follow up period, the participants will receive an information letter and a follow-up questionnaire after 6, 12 and 18 months. This questionnaire will be completed at home. Non-responders will receive a reminder after two and four weeks and responders who return an incomplete questionnaire will be re-contacted to complete the questionnaire by telephone.

We will invite approximately 20-25 participants to participate in focus group meetings. Each group meeting will consist of 7-8 persons. To ensure broad representation of the study population and to maximize the exploration of different perspectives, we will apply purposive sampling, based on age, gender, education level, severity of joint pain and sites of joint pain [32].

### Outcome measures

**Figure 3:**
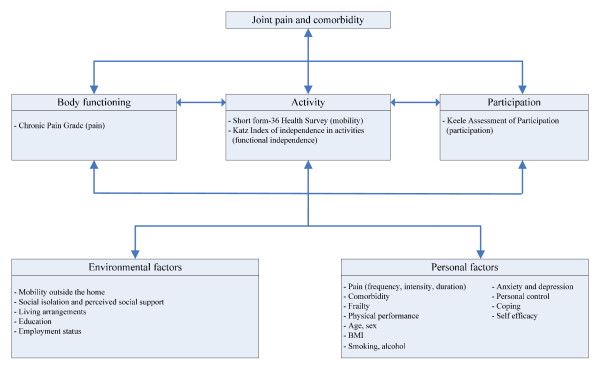
**Measurements, based on the ICF model (WHO, 2001)**.

We used the International Classification of Functioning, Disability and Health (ICF) as a framework for the measurements, as shown in Figure 3. The ICF model is a bio psychosocial model that is particularly suited for this study, because it emphasizes the importance of studying health problems from different perspectives. It has an integrated focus on somatic and social components of health and describes three levels of functioning, i.e. body functions (e.g. joint pain), activities (e.g. walking) and participation (e.g. participation in social activities), which can all be influenced by personal and environmental factors (i.e. possible predictors of the functional outcomes) [33].

#### Functional outcomes

Functioning will be assessed with self-report questionnaires at baseline and after 6, 12 and 18 months, in terms of mobility, functional independence and participation, by using questionnaires (table 2).

*Mobility *will be measured with the physical functioning subscale of the MOS 36-item Short Form Health Survey (SF-36), which consists of 10 items measuring difficulties in a hierarchical range of activities (e.g. vigorous activities, moderate activities, climbing several stairs, climbing one stair, walking more than one mile, walking several blocks) and can be scored on an ordinal 3-point scale (severe limitations, some limitations, no limitations) [34]. The Dutch version of the SF-36 (RAND-36) has proven to be reliable and valid in the older population [35].

*Functional independence *will be measured with the KATZ index of independence in activities of daily living [36], which measures the ability of the respondent to perform 8 ADL (e.g. bathing, dressing, toileting, walking, eating) and 7 IADL tasks (e.g. travelling, shopping, preparing meals, doing housework). Respondents can answer on a dichotomized scale (independent/dependent).

*Participation *restriction will be measured with the Keele Assessment of Participation questionnaire (KAP) [37], which contains 11-items: mobility inside the home, mobility outside the home, self-care, looking after belongings, looking after home, looking after dependants, interpersonal interactions, managing money and participation in work, education and social activities. Items capture performance, individual judgement and the nature and timeliness of participation (e.g. "*During the past four weeks, I have moved around my home, as and when I have wanted*") [37]. Responses are on an ordinal 5-point scale (i.e. all, most, some, a little, none of the time). The KAP has proven to be valid and reliable in the older population [37].

#### Quality of life

QoL will be measured at baseline and after 6, 12 and 18 months with one item of the SF-36, (*"In general, how would you rate your quality of life?") *on a 5-point scale *(*excellent, very good, good, reasonable, bad) [34]. Additionally, we will use Cantril's self-anchoring ladder for the evaluation of QoL [38].

#### Predictors of functioning and QoL

For the development of the prediction models, we will assess possible predictors of functioning and QoL at baseline, like pain, comorbidity, markers of frailty, physical performance, psychological factors, environmental factors and individual factors. Some time-dependent determinants will also be measured during the follow up period, such as psychological factors and living arrangements. Details are described in table 2 and Figure 3.

#### Health care utilization and health care needs

To study health care utilization, we will obtain data on health care use in general and health care use for joint pain in particular, as shown in table 2. Subjective health care use and health care needs will be assessed with the Camberwell Assessment of Need for Elderly People (CANE) in a face-to face interview. Additionally, we will explore health care needs in focus group meetings.

#### Meaning and impact of joint pain

To explore the personal experience and impact of joint pain in an older adult's everyday life, we will organize focus group meetings. Focus group meetings allow participants to share experiences and thus, enable exploration of the impact of joint pain, how joint pain interacts with other health problems, how people manage to lead their life despite pain and other health problems, which self management strategies they may use and how they could be supported by health care practitioners [32]. Participants can interact with each other, which makes it possible to clarify statements and opinions and allows investigation into beliefs, attitudes, behaviour and needs [39].

### Sample size

One of the main objectives of the study is to develop prediction models for poor functional outcome. This analysis demands the most statistical power and therefore, we calculated the sample size based on this analysis. Altman suggests using at least 10 'events' (i.e. older persons with deterioration in functional outcome) per predictor in a multivariable model [40]. Based on previous research we expect to find around 6-10 predictors [8,24,41] of poor functional outcome and this indicates the need of at least 100 participants with poor functional outcome after 18 months. We expect poor functional outcome in one quarter of the study population [42], which means that we need approximately 400 participants for the development of the prediction models. Because of possible loss-to-follow-up (10-15%), we aim to include 450 participants at baseline.

## Data Analysis

### Development of prediction models for functioning and QoL

We will use Latent Class Growth Mixture Modelling (LCGMM) to study functioning and quality of life over time [43]. Based on the ICF model, we will measure functioning from different perspectives, in terms of mobility, functional independence and participation. We assume that these functional outcomes are related to each other, because they all measure different aspects of functioning and provide information about functional status. Therefore, for functioning, the LCGMM consists of three steps. In the first step, within a Structural Equation Modelling framework, the three observed functional outcomes will be aggregated into one construct 'functioning' at each time-point. In the second step, for each individual the development over time of this construct functioning will be summarised into latent growth curve parameters (i.e. intercept, slope and if necessary quadratic slope). In the final step, individuals with comparable latent growth curve parameters will be grouped into clusters. The optimal number of clusters will be determined by using, among others, the Bayesian Information Criterion (BIC) and the bootstrap likelihood ratio test (BLRT) [44]. Depending on the number of clusters found by LCGMM, either binary (two clusters) or multinomial (more than two clusters) logistic regression will be used to predict different trajectories of functioning. We expect to find at least three clusters for functioning, i.e. improvement, no change (stable) or deterioration in functioning and think that it is clinically relevant to compare the contrasts between trajectories in the following two ways, i.e. improvement versus stable and deterioration versus stable. We will use univariate logistic regression analysis to study baseline characteristics that are associated with these different trajectories of functioning. Baseline characteristics that are strongly associated (p < 0.10), will be entered into a backwards stepwise logistic regression analysis to produce multivariate prediction models. The reliability of the model will be determined by plotting the predicted probabilities of poor functional outcome against the observed frequencies in a calibration plot and by calculating the C-statistic (discrimination) [37]. Bootstrapping will be used to correct for possible over-optimism of the model in our study cohort. This contributes to presenting a more precise estimate of the performance of the model [37].

We expect to find different predictors for QoL, and therefore QoL will be analysed separately by using LCGMM and logistic regression analysis.

### Health care utilization and health care needs

Objective health care utilization will be described by using descriptive statistics. Frequency tables will be used to summarize health care needs on the different items of the CANE and the extent to which these needs are met or unmet. We will use a paired T test or a Wilcoxon signed rank test to analyse differences between the amount of received and desired health care, to quantify unmet needs. To study the relation between sociodemographic characteristics and health care needs, we will use the chi-square test or the t-test, depending on the variables under study. Further data on health care needs will be collected in focus groups. The processing of the focus groups is described below.

### Meaning and impact of joint pain

The results of the focus groups will provide additional qualitative information on subjective health care needs and the meaning and consequences of joint pain in everyday living. This mixed methods approach enables us to generalise the quantitative results to the target population, while emphasising the personal perspective and experiences of older adults with joint pain and comorbidity. The results of the focus groups will be audio recorded and transcribed. Two independent researchers will code and group the data into categories for the identification of key points, by using ATLAS.ti. After the coding process, the two researchers will compare and discuss the categories, in order to achieve consensus.

## Discussion

This protocol describes a prospective observational cohort study in which extensive information will be gathered about older adults with joint pain and comorbidity. Previous studies indicate that older adults with joint pain and comorbidity have a higher risk of poor functional outcome, as joint pain is often poorly recognized and treated in primary care. Therefore, the main purpose of the study is to explore functioning in older adults with joint pain and comorbidity, in terms of mobility, functional independence and participation and to develop prediction models for the early identification of subgroups at high risk of poor functional outcome. Furthermore, the study will identify predictors of QoL. Early identification of older adults at high risk of poor functional outcome and decreased QoL may facilitate appropriate recognition, assessment, and treatment of joint pain, resulting in more effective and efficient primary care for this population and maintenance of functioning in daily living.

To strengthen our data collection and assimilation, we decided to extend our data resources by using various measuring methods. Besides quantitative methods for the exploration of functioning and QoL, the study design incorporates qualitative methods for the exploration of health care needs and the experiences and meaning of joint pain from the perspective of older adults, by organizing focus groups. The use of various measuring methods provides a more comprehensive understanding of joint pain in the older population, because the qualitative data are complementary to the quantitative data. The qualitative data further clarifies possible strengths and weaknesses in health care, and problems older people deal with in everyday life, which helps to optimize health care for this defined group. Another strength of this research protocol is that we incorporate the opinion and experiences of our target population into the different stages of the project. Two older adults who represent the target population have been recruited as members of the project team. They will be involved in the entire process and will provide input in several phases of the project, including the design and content of the information leaflet, letters and questionnaires and the objectives of the focus group meetings. We will also involve them in the presentation and dissemination of the results, in order to make sure that the results are clearly written and accessible for the target population, and all relevant professionals and stakeholders. A final strength is our selection procedure. We select patients based on self-report questionnaires for joint pain, instead of the medical records of the GP. The reason that we decided to screen on joint pain was because only a minority of the older people consults their GP for joint pain. Selection based on medical records would result in an underestimation of the number of people with joint pain and would provide a population sample which is not representative for the actual prevalence of joint pain in the older population.

However, this selection procedure also has a limitation. Screening with self-report questionnaires raises the possibility that people answer the questions about pain problems other than joint pain, like nerve pain or muscle pain, which could result in incorrect selection of people with joint pain. To minimize this problem, participants are asked to clarify their joint pain problems during the telephone call that we make when scheduling the appointment for the baseline assessment. Another limitation is the short follow up period of 18 months. Some earlier studies recommend longer follow-up periods, because of the relatively small changes in functioning after 2 years [45,46]. However, in our study population with participants older than 65 years and several limitations, we expect to find clinically relevant changes within 18 months.

In conclusion, the aim of the study is to obtain more insight into functioning and the different patterns of functioning and QoL in older adults with joint pain and comorbidity. This provides the opportunity to understand the differences in functioning and helps to identify possible risk factors that are associated with poor functional outcome and decreased QoL in the study population. Eventually, this could contribute to better recognition, assessment and treatment of joint pain and to more effective health care for older adults with joint pain and comorbidity.

## Competing interests

The authors declare that they have no competing interests.

## Authors' contributions

LH wrote the paper. SL has co-written the paper. DW designed the study and co-wrote the paper. MS has assisted in the design of the study and co-wrote the paper. JD has assisted in the design of the study and co-wrote the paper. HH has assisted in the design of the study and co-wrote the paper. All authors have been involved in revising the paper and have approved the final version.

## Pre-publication history

The pre-publication history for this paper can be accessed here:

http://www.biomedcentral.com/1471-2474/12/241/prepub
